# Reticuloruminal Motility Monitoring for the Prediction of Peripartal Hypocalcemia in Cattle

**DOI:** 10.3390/ani15223306

**Published:** 2025-11-17

**Authors:** Julia Gleissenberger, Philipp Breitegger, Matthias Gleissenberger, Michael Astl, Daniel Eingang, Georg Terler, Mathias Petermichl, Johann Gasteiner, Thomas Wittek

**Affiliations:** 1 University Clinic for Ruminants, Department for Farm Animals and Veterinary Public Health, University of Veterinary Medicine Vienna, Veterinärplatz 1, A-1210 Wien, Austria; julia.gleissenberger@vetmeduni.ac.at (J.G.);; 2 smaXtec Animal Care GmbH, Sandgasse 36/2, A-8010 Graz, Austria; philipp.breitegger@smaxtec.com (P.B.); michael.astl@smaxtec.com (M.A.);; 3 Institute of Livestock Research, Agricultural Research and Education Centre Raumberg-Gumpenstein, Alt-Irdning 11, A-8952 Irdning-Donnersbachtal, Austria; daniel.eingang@raumberg-gumpenstein.at (D.E.); georg.terler@raumberg-gumpenstein.at (G.T.); 4 Federal Agricultural Research and Education Centre Raumberg-Gumpenstein, Raumberg 38, A-8952 Irdning-Donnersbachtal, Austria; johann.gasteiner@raumberg-gumpenstein.at

**Keywords:** reticuloruminal motility, periparturient hypocalcemia, bolus wireless sensor, prediction, deep learning

## Abstract

Hypocalcemia commonly affects dairy cows around calving or at the onset of lactation. An individual risk prediction would allow customized prophylactic measures and targeted intervention. This study aimed to measure concentrations of total (tCa) and ionized calcium (iCa) during the peripartal period, record rumen motility and temperature patterns, and assess the associations between patterns before calving and Ca concentration at calving. A total of 89 calvings from 47 cows and 22 heifers were monitored using reticuloruminal bolus sensors over 60 days prior to the expected calving and 60 days postpartum. Cows with low tCa (<1.8 mmol/L) or iCa (<0.8 mmol/L) a few days before parturition had reduced rumination times compared to cows with normal tCa levels (>2.2 mmol/L). Using a rumination time (RT) threshold of 480 min/day on day −1, hypocalcemia was predicted with 66.7% sensitivity and 96.8% specificity. Additionally, we evaluated a deep learning model trained on external data, which incorporated rumen cycles, reticular contraction duration, and reticular temperature. Despite not being trained on this dataset, the model surpassed the RT thresholding approach, achieving 83.2% sensitivity and 98.2% specificity for tCa-based classification. These results indicate superior performance and greater generalizability of the deep learning approach, highlighting the potential of multi-metric sensor analytics to improve early hypocalcemia risk detection.

## Simple Summary

Currently, cow-level prediction methods of hypocalcemia risk, which are based on laboratory results, have only low to moderate predictive values. The present study explores whether data measured by a reticuloruminal bolus wireless sensor may be used for the prediction of hypocalcemia risk before or close after calving. Cattle fitted with rumen sensors were monitored during the peripartal period, and blood samples were obtained for laboratory analyses. The sensor continuously recorded rumination time, rumen motility, and reticular temperature. Correlations between total serum calcium concentrations at parturition and variously, rumination time, reticular temperature, as well as the rumen motility during the days before calving have been found, indicating a potential predictive value of these latter parameters. Furthermore, the data collected in this study were used to assess the prediction quality of a deep learning model, which relies on rumen cycles, reticular contraction duration, and reticular temperature. The model has not been trained on the data of the present study, but it outperforms a simple approach based on rumination time.

## 1. Introduction

Hypocalcemia, also known as parturient paresis or milk fever, typically occurs in cattle during calving at the start of lactation. This disease is characterized by muscle weakness, paralysis, recumbency, and impaired function of the central nervous system [1]. If the animal is not treated accordingly, it may lead to muscle damage or even death [2,3]. To confirm the clinical diagnosis based on physical examination, the serum or plasma calcium concentration is frequently analyzed. The total calcium concentration (tCa) in the serum is typically measured photometrically; the ionized calcium (iCa) can be measured on site using an ion-selective electrode as a point-of-care test. Earlier studies suggested that the proportion of iCa is 50% of tCa [4], but this proportion is decreased around parturition [5].

Over the years, extensive research has been carried out on the pathogenesis and treatment of periparturient hypocalcemia. Depending on the literature, the prevalence of clinical hypocalcemia lies between 5% [6] and up to 10% especially on high-producing farms [7,8]. There are several factors that influence the occurrence of hypocalcemia; one factor is high milk yield [9]. A cow that produces around 45 kg of milk per day on average needs an additional 56 g/d of Ca [10]. A non-lactating cow requires approximately 21 g of calcium per day to maintain normal body functions [10]. Furthermore, with increasing lactation number, the risk of hypocalcemia increases substantially [9]. The study from [6] showed a significant decrease in serum calcium concentrations as the number of lactations increased from 1st to 4th lactation. According to DeGaris and Lean [8], this reflects the effects of age and results in a 9% increased risk of hypocalcemia per lactation. Generally, dairy breeds are more susceptible than beef breeds [8]. Another factor for an increased risk of hypocalcemia is over-conditioned cows (body condition score > 3.5) [9]. In addition, various other factors, such as the length of the dry period, may additionally influence hypocalcemia risk [11].

The digestive physiology of ruminants has been extensively researched in recent decades, as forestomach motility can be used as an overall indicator of cattle health [12]. Reticuloruminal motility (RRCR) refers to the complete sequence of coordinated contractions in the reticulum and rumen, consisting of the A (primary) and B (secondary) cycles [13,14]. The A cycle represents the biphasic reticular contraction followed by ruminal contractions that mix and move digesta between the reticulum and rumen, while the B cycle involves additional ruminal contractions responsible mainly for eructation (gas expulsion). During rumination, extrareticular contractions occur, complementing these rhythmic cycles [13,14]. In general, forestomach motility, as with all muscle activity, depends on serum calcium concentration. Low calcium concentrations, which are common after parturition, have negative effects on the rumen motility and rumination time [15]. The frequency and amplitude of RRCR increase transiently during feeding and decrease during rumination and lying [14]. A rumination cycle lasts about one minute [13]. After swallowing the bolus, it takes a few seconds before a new rumination cycle begins [13]. On average, healthy dairy cows spend around seven hours a day ruminating [2], and four and a half hours (2.4–8.5 h) eating [16]. Cattle that have constant access to feed spend more time ruminating and less time eating [16].

In general, forestomach motility, as with all muscle activity, depends on serum calcium concentration [15]. Low calcium concentrations, which are common after parturition, have negative effects on the rumen motility and rumination time [15]. There are commercially available devices (In-Cow and On-Cow sensors) that can monitor the rumination time of individual animals. One device for measuring reticuloruminal motility is a reticuloruminal bolus, which constantly measures motility and temperature inside the reticulorumen.

First, this study aimed to measure rumen motility and temperature by reticuloruminal bolus before, during, and after parturition and to associate the measurements with the peripartal serum concentration of total and ionized calcium. Second, the study aimed to assess the feasibility of prepartum rumen measurements to predict the hypocalcemia risk at parturition on the individual cow level. This approach addresses the current limitation that existing cow-level prediction methods based on laboratory biomarkers (total serum calcium, magnesium, phosphorus, non-esterified fatty acids, β-hydroxybutyrate, alkaline phosphatase) tend to offer only low to moderate predictive accuracy [17,18,19].

The following hypotheses were proposed:

1. The reticuloruminal contraction and reticoluruminal temperature patterns before calving are associated with the periparturient calcium concentration.
2. Pre-calving reticuloruminal motility and temperature measurement is feasible to predict the risk of periparturient hypocalcemia at the individual cow level.
3. The deep learning model, which uses reticuloruminal sensor data as features, can reliably predict the hypocalcemia risk for the data of this study without having been trained on them.

## 2. Materials and Methods

The study was conducted at the Agricultural Research and Education Center (AREC) Raumberg-Gumpenstein (Irdning-Donnersbachtal, Austria) between November 2022 and March 2024. During this study period, 89 calvings of 69 cattle were recorded (22 calvings of first calf heifers, 47 calvings of first to third lactation, and 20 of fourth to tenth lactation cows). The average age of the cows at calving was 4.4 ± 2.3 years, the youngest heifer was 1.9 and the oldest cow was 11.5 years old. The breed distribution was 20 Fleckvieh (Simmental), 39 Holstein-Friesian, and 10 New Zealand Holstein-Friesian cows. All cows were housed in a free stall barn with separate feeding troughs and an automated milking system. The barn is equipped with a curtain system, ventilation, and sprinklers to allow temperature adjustment. Until 14 days before the expected calving date, the cows were fed twice daily with a dry cow diet consisting of a partially mixed ration. After that point, the cows were given a lactation diet ad libitum.

The farm administers a vitamin D3 injection to all cows entering their third lactation before calving, which prevents any clinical cases of milk fever. However, during the study, this routine preventive measure was discontinued.

The cows were fed an additional amount of 1 kg/d of a commercial concentrate (Kuhkorn PLUS Energie^1^, Garant Tiernahrung GmbH, Pöchlarn, Austria) during the last 14 days before expected calving. Diet composition and chemical analyses for the standard lactation and dry cow diet on a DM basis are presented in Table 1.

After showing signs of approaching birth (less feed intake, swelling of the udder, vaginal discharge, softening of the pelvic ligaments), the animals were moved to a calving pen with straw bedding. Depending on their health status after parturition, the cows were returned to the free stall after a few days.

For continuous data collection (data was recorded at 10 min intervals), the cattle were fitted with a reticulorumen sensor (classic bolus; smaXtec animal care GmbH, 8010 Graz, Austria) 60 days before the calculated calving date. The sensor weight is 210 g, the dimensions are 105 × 35 mm, and it is made of ruminal fluid-resistant plastic. The bolus that was used in the present study is commercially available and used on numerous farms for different purposes (e.g., heat detection, calving detection, early detection of diseases). The manufacturing company uses proprietary algorithms to calculate cow-specific time series data. For this study, the data on locomotion activity (LA), rumination time (RT), reticuloruminal motility (RRCR), and reticular temperature (RETT) were recorded between the time of 60 days antepartum until 60 days after calving.

The cows were physically examined at dry off (60 days before the calculated calving date), and the heifers approximately sixty days before the expected calving date. The physical examination recorded general behavior, respiration frequency, heart rate, color of the mucous membrane, internal body temperature, skin temperature, rumen motility, appetite, defecation, and urination [20]. All animals were repeatedly examined three (−21 d), two (−14 d), and one (−7 d) weeks before the expected calving, three to six hours before (−0 d), during (0 d), and one to four hours after the calving (+0 d). Further examinations were performed on day one (+1 d), three (+3 d), seven (+7 d), and fourteen (+14 d) postpartum (study setup is shown in Table 2). The physical examination was performed to ensure that all animals in the study were clinically healthy.

Blood samples were obtained from each animal during the physical examinations. Blood was collected via coccygeal venipuncture into Vacuette serum blood tubes (Greiner Bio-One GmbH, 4550 Kremsmünster, Austria). After sampling, the blood was left to stand at room temperature for approximately 20 min and then centrifuged (3000× *g* for 20 min). After centrifugation, the serum was transferred to Eppendorf Safe-Lock tubes and stored at −21 °C.

Since the animals did not always give birth on the expected day, the actual day of the blood sampling before parturition was recalculated according to the day of calving. Data from animals (*n* = 13) that gave birth to twins (*n* = 4), inadvertently received vitamin D3 prophylaxis (*n* = 6) or suffering from disease (respiratory diseases; *n* = 3) had been excluded from the analysis.

Immediately after centrifugation, the ionized calcium (iCa) was measured with the ion meter LAQUAtwin Ca-11C^®^ (HORIBA Advanced Techno, Kyoto, Japan) in the serum. Serum was used for analysis of calcium (tCa), phosphorus, alkaline phosphatase, magnesium, and potassium at the laboratory for clinical diagnosis, Laboklin (4020 Linz, Austria).

The categorization of the hypocalcemia groups was based on tCa and iCa samples taken on day +0. The cows were subdivided into healthy, subclinical hypocalcemic (SCH), and low-calcium (LC). Cows were retrospectively categorized based on serum tCa and recategorized based on serum iCa concentrations as healthy (tCa > 2.20 mmol/L; iCa > 1.05 mmol/L), subclinical hypocalcemic (SCH; 2.2 mmol/L > tCa > 1.80 mmol/L; 1.05 mmol/L > iCa > 0.80 mmol/L), or low-calcium (tCa < 1.80 mmol/L; iCa < 0.80 mmol/L) [6,9,19]. These thresholds were adapted from previously published biochemical definitions of calcium status [6,9,21], which are within the range of cutoff values commonly reported in the literature. The work by Neves et al. [19] was cited to illustrate the variability of classification approaches used in recent studies.

For the statistical analysis, the software programs R 3.0.2 (R Development Core Team, Vienna, Austria, 2013) as well as Python 3.11.11 with Pandas 2.2.3 and Plotly 6.2.0 were used. The dataset was imported, cleaned for missing or erroneous values, and converted into a data frame containing the variables: time, temperature without drinking cycles, forestomach motility, rumination time, tCa, and iCa. The arithmetic means of the sensor-based parameters (rumination activity, (delta) ruminal temperature, reticuloruminal contraction rate, delta average duration of rumen cycle net rate) were calculated for 12 h intervals from day −21 to +7. Data visualization was performed using the ggplot2 package in R 3.0.2 (R Development Core Team, Vienna, Austria, 2013). Histograms and Q–Q plots were used to assess normal distribution. Box plots were drawn of the three groups for comparison. To check the variance homogeneity, Levene’s test (leveneTest, car package) was performed on these parameters. For the analysis of variance of the three groups, the ANOVA test (aov) was used. The Bonferroni post hoc test (t.test, p.adjust.method = “bonferroni”) was used to calculate the days for which the mean values were significantly (*p* < 0.05) different between the groups. Repeated measures were analyzed iteratively across time points. The fixed effect in the model was Ca status (healthy, subclinical, low-calcium), while time was treated as a repeated factor. No random effects were included, as each time point was analyzed independently to assess temporal trends. For diagnostic evaluation, only cows with negative time values and without subclinical cases were included. The ROC (receiver operating characteristic) curve (cutpointr package [22]) is a graphical method for determining the performance of a diagnostic test and helps to find the optimal cut off point for differentiating between diseased and non-diseased animals. Youden’s J statistic, which is calculated as the maximum value of (sensitivity + specificity − 1), was used to differentiate between healthy and low-calcium cows. Odds ratios were calculated from contingency tables derived from the ROC classifications.

To assess the potential of more advanced analytics beyond simple thresholding of rumination time, which may miss subtle or nonlinear indicators of disease, a deep learning (DL) model provided by smaXtec was evaluated. This model is part of the TruAdvice ™ early detection system [23], which is currently used on commercial farms for early detection of milk fever risk in dairy cows.

The DL model is a binary classifier that predicts the risk of clinical hypocalcemia for individual cows. The underlying architecture is based on InceptionTime, a deep learning model originally developed for time series classification tasks [24]. InceptionTime uses a series of convolutional neural networks (CNNs) to identify and learn complex patterns over time within and across multiple features. While CNNs are often used in image analysis, they can also learn complex temporal patterns in longitudinal sensor data, such as those collected from reticuloruminal sensors.

The model was trained on data collected from diverse farms worldwide, representing a wide variety of breeds, environments, and management styles. Importantly, none of the data from this current study was used in the model’s training, validation, or testing phases, making our results a true external validation of the model’s generalizability. The model uses five features: four time-series features derived from the reticuloruminal bolus sensor, and one static feature indicating animal parity. The time-series features include:

- RETT (Reticular Temperature)
- RCNR (Rumen Cycle Net Rate, i.e., duration of A or A + B rumen cycle in seconds [14])
- RCD (Reticular contraction duration, i.e., length of biphasic or triphasic reticular contraction in seconds)
- RCDV (Percentage of successful RCD calculations)

Each feature is resampled to 10 min intervals, resulting in 144 data points per day. To predict the risk on a given day, the model uses a 12-day window. All features are standardized, and missing values are imputed using linear interpolation. Furthermore, the model includes the information whether the animal is a cow or heifer.

These features form the multivariate input time series processed by the DL model, as detailed below.

The model architecture consists of:

- 14 inception modules
- convolutional kernel sizes of 216, 108, and 54 (in each module)
- and a bottleneck layer producing 12 feature maps per module

This configuration yields approximately 773,000 trainable parameters. The model was trained using cross-entropy loss and the Adam optimizer with a batch size of 16. Data was split into training (72%), validation (18%), and test (10%) sets. Early stopping with a patience of 15 epochs without improvement in validation accuracy and dropout layers helped reduce potential overfitting. Hyperparameter tuning and threshold selection were guided by the precision–recall performance of the test set.

Implementation note: The model was implemented in PyTorch 2.3 and trained on an NVIDIA Tesla T4 GPU.

## 3. Results

**Figure 1:**
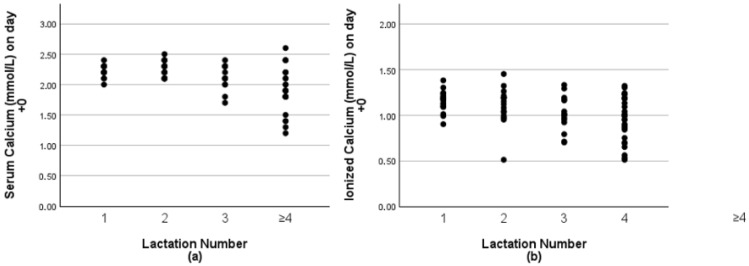
(**a**) Total serum calcium (tCa) concentrations and (**b**) ionized calcium concentrations of 20 heifers and 56 cows were measured, approximately three hours after parturition (+0). Lactation number 1 (*n* = 20), 2 and 3 (*n* = 36), and ≥4 (*n* = 20).

A total of 76 calvings from heifers and cows (20 heifers and 56 cows) were included in the analyses. Figure 1 shows the tCa (a) and iCa (b) concentrations after calving distributed according to the number of lactations.

Table 3 shows the distribution of the three groups: healthy, SCH, and LC.

The proportion of ionized calcium to total calcium increased sharply on the day of calving. On day −7, the average iCa:tCa ratio was 0.43 (±0.08), the average iCa:tCa ratio on day −0 was 0.48 (±0.08), and at calving it was 0.49 (±0.09) (Table 4).

### 3.1. Rumination Threshold Approach

**Figure 2:**
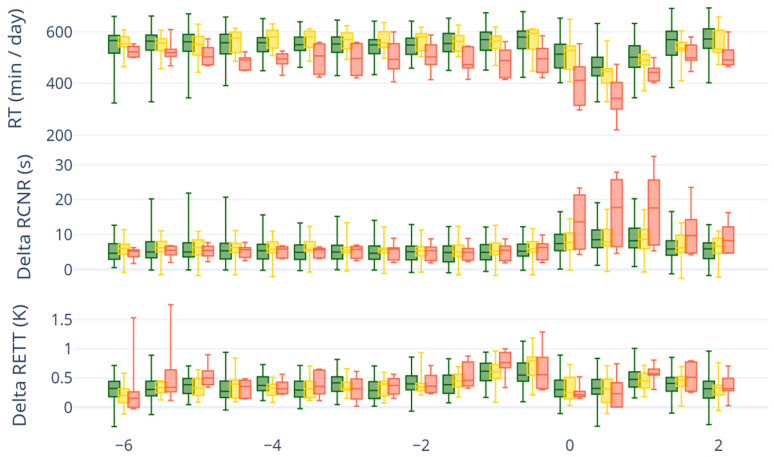
Box-plot presentation of the maximum value of RT, Delta RCNR, and Delta RETT grouped by healthy (tCa ≥ 2.2 mmol/L; green), subclinical hypocalcemia (tCa < 2.2–1.8 mmol/L; yellow), and low-calcium (tCa < 1.8 mmol/L; red) over time (0 indicates day of calving). Delta RETT and Delta RCNR values represent the deviation from the 24 h rolling mean of each respective variable. The length of the box corresponds to the area in which the middle 50% of the data lie. It is therefore limited by the upper and lower quartiles (25 and 75%).

The boxplots (Figure 2) show rumination time, Delta RCNR, and Delta RETT. Delta RETT and Delta RCNR values represent the maximum deviation from the 24 h rolling mean of each respective variable. Significant differences in RT were observed between low-calcium (LC) and healthy cows on several days around calving, specifically on days −4, −3.5, −3, −2.5, −1.5, −1, −0.5, 0, 0.5, 1, 2, and 2.5 (Figure 2). In addition, significant differences in RT were also detected between low-calcium (LC) and subclinically hypocalcemic (SCH) cows on days −4.5, −4, −3.5, −3, −2.5, −2, −1.5, −1, −0.5, 0.5, and 1.

The RETT itself did not show differences between the three groups. However, Delta RETT was higher on average for LC than for healthy cows on days −2.5, −1.5, −1, 1, and 1.5. In addition, differences can also be detected between LC and SCH cows on days −1.5, −1, 1, and 1.5. Furthermore, the number of temperature decrease events (Table A2 and Table A3) could be observed for LC cows at a larger percentage than for SCH and healthy cows.

Similarly, RRCR itself did not show significant differences between the three groups (classification based on tCa). However, Delta RCNR was higher on average for LC than for healthy cows on days −1, −0.5, 0, 0.5, 1, 1.5, and 2. The difference was highest on days 0, 0.5, and 1. Also, differences can be seen between LC and SCH on the same days.

Based on these results, RT was selected as the primary variable for the threshold-based model due to its stronger statistical significance for the data of the given study. While RT showed the highest statistical significance in this study, the results also underline the value of including RCNR and RETT as features in the deep learning model. Although their significance was lower in this cohort, previous external training has demonstrated that these variables generalize well across different farms, breeds, and management systems. No significant differences in RCD and RCDV were observed between the three classes in this study.

In contrast to the classification based on total calcium (tCa), the reticuloruminal parameters differed less if ionized calcium (iCa) was used for classification in healthy, SCH, and LC. Therefore, only tCa was used for classification. For day −1, which is the day with the highest predictive value, the optimal RT cut-off is 480 min/day (Table A1); this results in 66.67% sensitivity, 96.40% specificity, and an AUC of 0.87 (ROC curve analysis shown in Appendix A).

### 3.2. Model Evaluation on Study Cohort

The DL model was evaluated on the study cohort using a fixed prediction threshold of 0.75, which corresponds to the standard sensitivity setting used in practice. The model is evaluated every hour to allow for timely notification.

**Figure 3:**
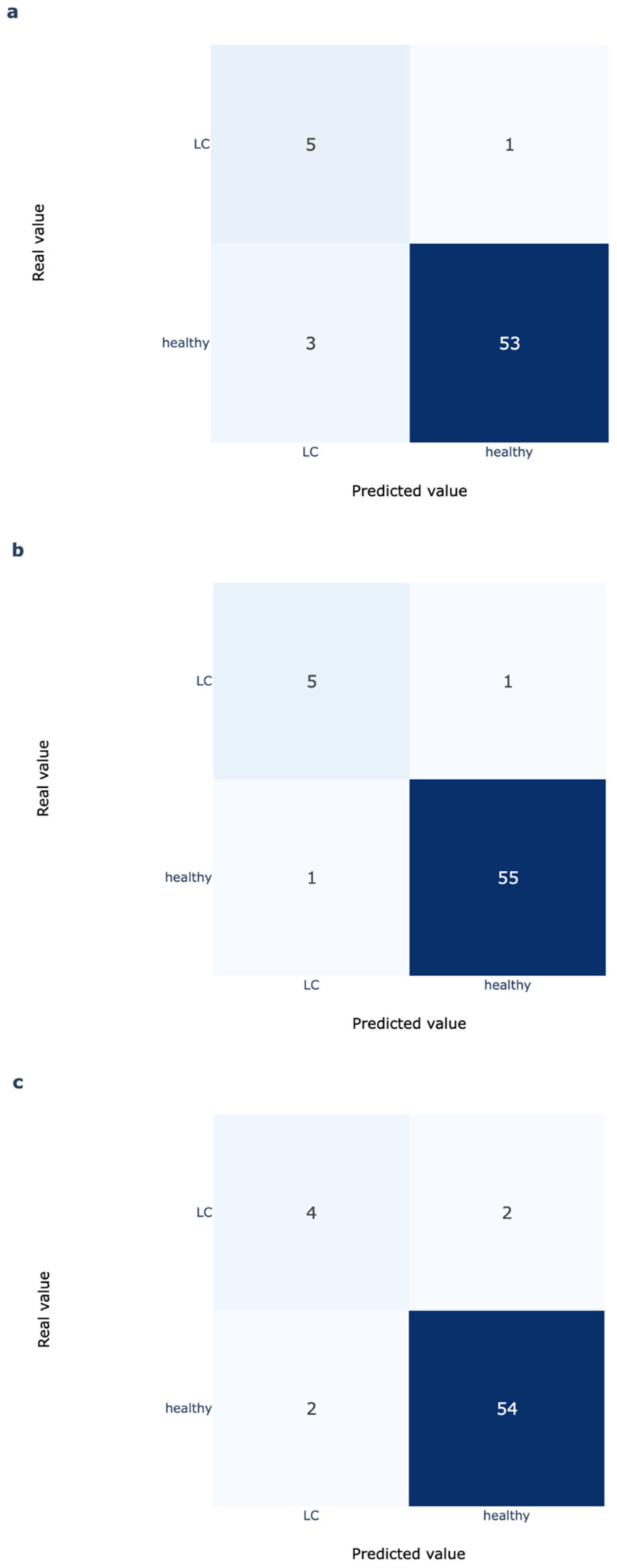
Confusion matrices for DL Model with threshold 0.75 (**a**) and 0.9 (**b**) until 2 h postpartum and RT model at day −1 day antepartum (**c**).

For this setting, the model was able to detect five out of six LC cows with only three out of 56 false positives (Figure 3). This is equal to a sensitivity of 83.2% and a specificity of 94.6% for the classification based on tCa. A value of 0.90 was calculated for the AUC. The positive predictions were triggered between 12 h antepartum and 1 h postpartum, with a mean value of 4 h antepartum. When the threshold was increased to 0.9, specificity improved to 98.2% with the same sensitivity, and the average time of positive prediction was still 1 h antepartum.

In contrast, the RT thresholding approach detected only four out of six LC cows with two out of 56 false positives. This is equivalent to a sensitivity of 66.7% and a specificity of 96.4%. These results demonstrate that the DL model identifies more at-risk cows without sacrificing specificity.

## 4. Discussion

### 4.1. Prevalence and Biomarker Interpretation

The proportion of cows with low calcium concentrations in our study cannot be directly compared to the 7% incidence of clinical hypocalcemia reported by Venjakob and Borchardt [9], as our classification was based solely on serum calcium values without clinical assessment. While acknowledging that more dynamic, outcome-associated definitions (e.g., [25,26]) could enhance biological interpretation, we recognize that implementing such models would necessitate longitudinal calcium measurements and health outcome data, which are not included in the current dataset. It was also stated that one in two dairy cows developed subclinical hypocalcemia during the peripartal period. The present study showed similar results 51.32% had subclinical hypocalcemia [9,19]. The hypocalcemia classification system used in this study [9] was the most appropriate, as using different thresholds for calcium concentrations would have resulted in slightly different predictive values. The small number of hypocalcemia cases made it impractical to use a system that characterized additional categories [25,27].

As expected, no heifers developed hypocalcemia and only 9 showed subclinical hypocalcemia (tCa 1.8–2.2 mmol/L). In contrast, 5 out of 6 cows in their ≥4th lactation were LC (tCa < 1.8 mmol/L). Reinhardt et al. [6] described the effects of age and the associated increase in the number of cows with subclinical hypocalcemia. Despite the small number of animals in this study, these results followed the same pattern.

Measuring iCa within the first four days after calving may provide information on calcium homeostasis in the herd [10]. However, total serum calcium is more stable than ionized calcium, and until recently, it was also easier and less expensive to measure. [10]. Arnold et al. [28] analyzed several antepartal laboratory parameters (total serum calcium, ionized calcium, alkaline phosphatase, and net acid-base excretion) for predicting postpartum hypocalcemia. They found no significant correlation between antepartum iCa and postpartum tCa and concluded that none of the parameters studied could be used reliably to predict hypocalcemia. The literature describes the measurement of iCa with ion ion-selective electrode as more complex [29,30]. This is due to the fact that the temperature of the whole blood should be 37.0 °C, the contact with air must be minimized, the use of the correct anticoagulant, and the pH should be adjusted [4,5,29]. In this study, ionized calcium was measured in the serum immediately after centrifugation. Consistent with the findings of Ott et al. and Leno et al. [5,31], we observed changes in the concentration of iCa in the peripartal period. In the current study, iCa:tCa ratio increased in the last three weeks before calving from 0.39 on day −21 to 0.43 on day −7 to 0.48 on day −0. However, iCa:tCa did not change after calving until the end of the measurements (day +14; iCa:tCa 0.48). In the study from Ott et al. [5], the iCa:tCa ratio returned to 0.43 within 7 days after calving, but the number of 14 cows is rather small to make a significant statement. These results contradict the previous assumption that iCa makes up about 50% of the total calcium in the blood of cows [4]. Some studies consider iCa a superior parameter for the detection of clinical and subclinical hypocalcemia in comparison to tCa concentration [10]. In the present study, tCa had a higher predictive value. Given the small numbers here, further research is needed to determine the practical usefulness of iCa in serum. It is to be mentioned that it seems there is currently no validation for the LAQUAtwin Ca-11C^®^ device for serum. This is also one of the reasons why total Ca was used in the final model.

### 4.2. Deep Learning-Based Risk Prediction

It is claimed by smaXtec that the limitations of an RT threshold approach, as observed here, can be addressed using deep learning (DL) models that integrate multiple features from intraruminal boluses and are designed to generalize across farms, breeds, and countries. DL models can be used to identify and learn complex patterns over time within and across multiple features to predict hypocalcemia risk. If trained on a large enough, balanced dataset, they can identify patterns that extend beyond the conditions of any single farm.

In this study, we evaluated a DL classifier developed by smaXtec, trained on data from multiple international farms. Unlike the RT model, the DL classifier does not rely on knowledge of the calving date or a single feature; instead, it uses a 12-day time series window across four sensor-derived parameters—RETT, RCNR, RCD, and RCDV—processed through a network composed of multiple convolutional neural networks. Although the smaXtec deep learning model has not been trained on the data presented in this study, it outperformed the simple approach with a sensitivity of 83.2% and a specificity of 98.2% compared to 66.67% sensitivity and 96.40% specificity

van Leerdam et al. [32] reported that their best-performing hypocalcemia risk prediction model was based on Long Short Term Memory (LSTM) based networks, incorporating 21 days of behavioral sensor data from neck and leg devices along with static features such as parity, season, and calcium measurement day. In contrast, the smaXtec DL model relies exclusively on reticuloruminal bolus data (RETT, RCNR, RCD, RCDV) plus parity yet achieves a substantially higher AUC of (0.90 vs. 0.71) and average precision (0.83 vs. 0.47). These results highlight the predictive value of reticuloruminal parameters for hypocalcemia detection.

## 5. Conclusions

Both models offer novel approaches to assessing the risk of hypocalcemia before parturition on an individual cow level as prediction tools. The ability to identify cows at-risk up to 12 h before calving offers the opportunity to implement preventive measures. Veterinarians and farmers have the option of administering targeted calcium supplementation or adjusting feeding strategies to stabilize calcium metabolism before the onset of clinical symptoms. The ability to reliably predict hypocalcemia at the individual cow level—particularly in small herds—holds considerable value. In such herds, group-level prophylaxis is often impractical, making individualized risk prediction highly attractive. The findings from this study support the integration of reticuloruminal sensor-based analytics, especially deep learning models, into proactive health management strategies.

Our results show that a simple rumination time (RT) threshold can provide moderate predictive accuracy for hypocalcemia. However, the deep learning models trained on large, diverse datasets provide superior performance and broader applicability. Future work should focus on extending the model framework, possibly including other sensor data to detect additional conditions, combined with field studies for verification.

## Figures and Tables

**Table 1:** Composition and chemical analysis of the dry cow diet (from dry-off at ±60 days before expected calving until 14 days before expected calving) and lactation diet fed to dairy cows—antepartum and postpartum (starts 14 days before expected calving), as well as of commercial concentrate.

	Dry Cow Diet	Lactation Diet	Commercial Concentrate ^1^
**Ingredient (% of dry matter)**			
Grass hay	50.0	32.5	
Corn silage		25.0	
Grass silage	50.0	42.5	
**Chemical analysis (g/kg dry matter, unless stated; *n* = 18 per feed) ^2^**			
Dry matter, g/kg fresh matter	488.4 ± 69.8	400.7 ± 54.7	908.5 ± 15.7
Crude protein	156.4 ± 16.2	144.8 ± 13.7	172.0 ± 6.5
Crude fat	26.1 ± 4.6	28.4 ± 4.2	35.9 ± 3.5
Neutral detergent fiber	509.5 ± 38.7	498.1 ± 37.5	175.1 ± 13.4
Calcium (Ca)	7.2 ± 0.9	6.3 ± 1.0	8,7 ± 0.4
Phosphor (P)	3.2 ± 0.3	3.1 ± 0.2	5.3 ± 0.3
Magnesium (Mg)	3.1 ± 0.5	2.8 ± 0.4	3.0 ± 0.2
Potassium (K)	24.4 ± 2.6	22.2 ± 1.5	8.6 ± 0.6
Sodium (Na)	0.6 ± 0.2	0.5 ± 0.2	2.5 ± 0.2
Zinc (Zn), mg/kg dry matter	32.2 ± 4.9	29.3 ± 3.5	119.2 ± 7.4
Copper (Cu), mg/kg dry matter	10.2 ± 1.5	9.1 ± 1.0	22.7 ± 0.9

^1^ The mineral mix contained per kg: 6% calcium carbonate; 10% phosphorus; 12% sodium chloride; 5% magnesium oxide; 800,000 I.U. vitamin A; 80,000 I.U. vitamin D3; 2500 mg vitamin E; 350 mg iodine (calcium iodate); 50 mg cobalt (coated cobalt (II) carbonate granules); 1200 mg copper (copper (II) sulfate pentahydrate); 4000 mg g manganese (manganese (II) sulfate monohydrate; 6000 mg zinc (zinc oxide); 50 mg selenium (sodium selenite). ^2^ The chemical composition was analyzed in samples taken at monthly intervals from November 2022 to March 2024. Values are presented as Means ± Standard Deviation.

**Table 2:** Timeline of physical examination, sampling, measurements, and data recording.

Measures	Day	Timepoint
Physical examination, insertion of the sensor, start of data recording	60 days before the expected calving	−60
Physical examination, blood sampling	21.9 ± 5 days before the calving	−21
Physical examination, blood sampling	14.6 ± 4.7 days before the calving	−14
Physical examination, blood sampling	7.8 ± 4.5 days before the calving	−7
Physical examination, blood sampling	Day of calving, a few hours (2.8 ± 1.6 h) before calving	−0
Physical examination, blood sampling	Calving	0
Physical examination, blood sampling	Day of calving, a few hours after calving (2.8 ± 1.6 h)	+0
Physical examination, blood sampling	1 day after calving	+1
Physical examination, blood sampling	3 days after calving	+3
Physical examination, blood sampling	7 days after calving	+7
Physical examination, blood sampling	14 days after calving	+14
End of data recording	60 days after calving	+60

**Table 3:** Distribution of cows by calcium concentration (tCa and iCa) postpartum.

	*n*	%	Lactation Number		
**Total Serum Calcium**			**1**	**2–3**	**≥4**
Healthy (≥2.2 mmol/L)	31	40.79	11	17	3
SCH (<2.2–1.8 mmol/L)	39	51.32	9	18	12
LC (<1.8 mmol/L)	6	7.89		1	5
**Ionized serum calcium**					
Healthy (>1.05 mmol/L)	41	55.26	17	20	4
SCH (≤1.05–0.8 mmol/L)	24	30.26	3	14	7
LC (<0.8 mmol/L)	11	14.48		2	9

**Table 4:** Time-dependent changes in ionized calcium concentration (iCa) measured from serum, total serum calcium concentration (tCa), and the resulting ratio iCa:tCa.

Sampling Day	iCa (mmol/L)	tCa (mmol/L)	Ratio iCa:tCa
−21	1.17	2.97	0.39
−14	1.16	2.93	0.40
−7	1.16	2.77	0.43
−0	1.11	2.26	0.48
0	1.07	2.16	0.49
+0	1.06	2.12	0.49
+1	1.05	2.09	0.49
+3	1.13	2.29	0.48
+7	1.15	2.42	0.47
+14	1.17	2.43	0.48
SEM	0.06	0.12	0.03

The values used were averaged, and the pooled standard error of the mean (SEM) values were calculated. iCa = ionized calcium measured in the serum after centrifugation, tCa = total serum calcium.

**Figure A1:**
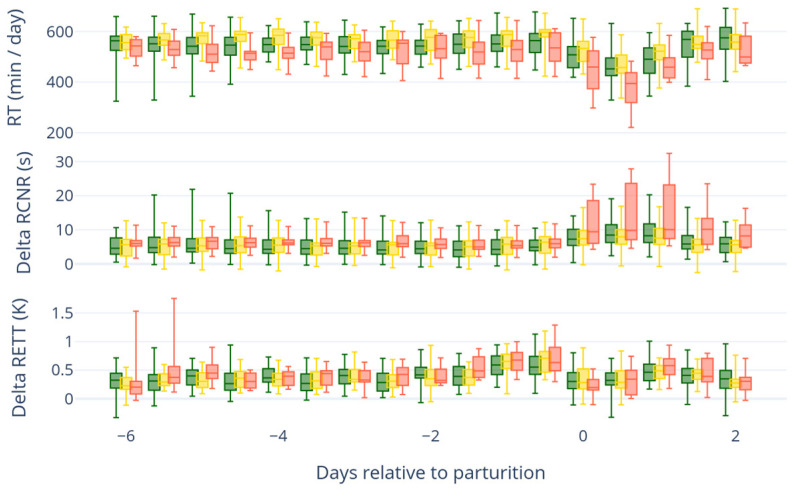
Box-plot presentation of the maximum value of RT, Delta RCNR and Delta RETT grouped by healthy (iCa > 1.05 mmol/L; green), subclinical hypocalcemia (iCa 0.8–1.05 mmol/L; yellow), and low-calcium (iCa < 0.8 mmol/L; red). Delta RETT and Delta RCNR values represent the deviation from the 24 h rolling mean of each respective variable. The length of the box corresponds to the area in which the middle 50% of the data lie. It is therefore limited by the upper and lower quartiles (25 and 75%).

**Figure A2:**
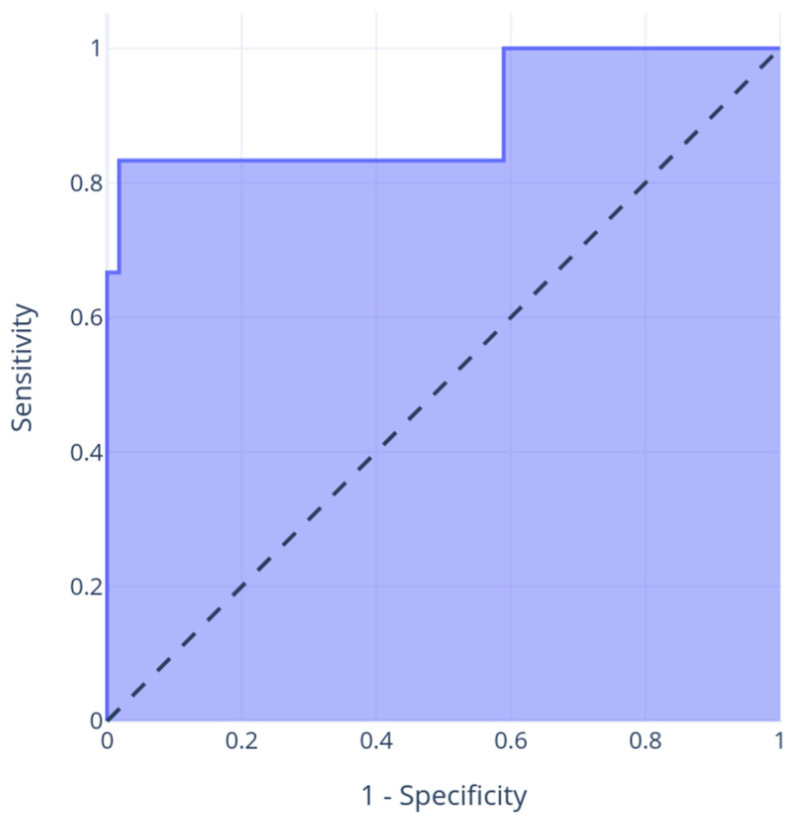
ROC curve of the Deep Learning Model.

**Figure A3:**
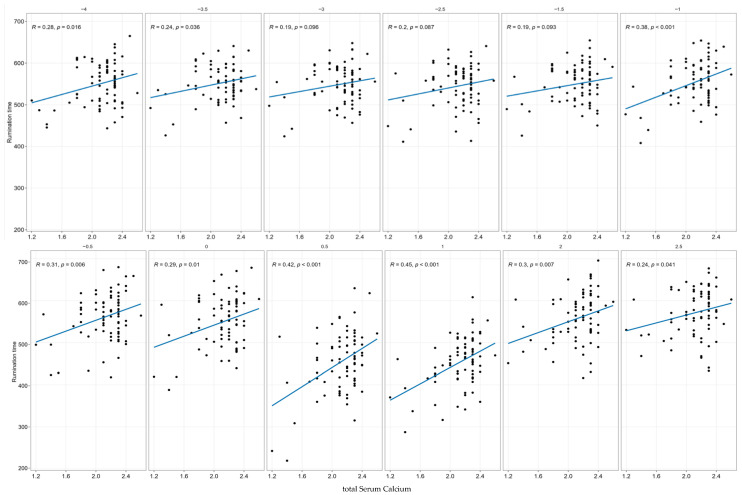
Correlation on *p*-values between peripartal tCa (mmol/L) and rumination time (min/day) for several days before and after parturition.

**Figure A4:**
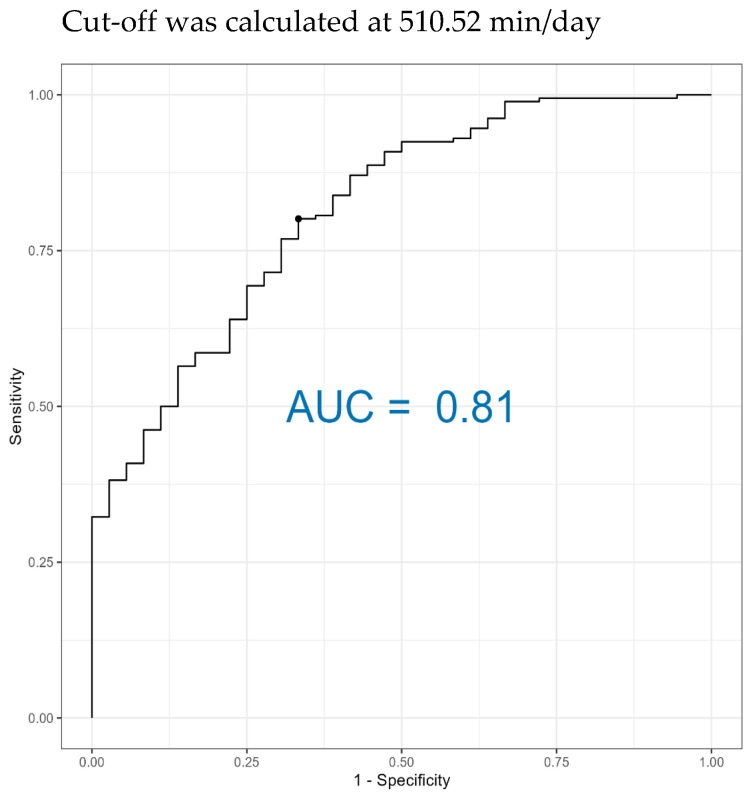
Mean ROC curve of RT of healthy and LC. The optimal cut-off was calculated at 510.52 min/day for the mean over all relevant days antepartum (−4, −3.5, −3, −2.5, −1.5, −1, −0.5).

**Figure A5:**
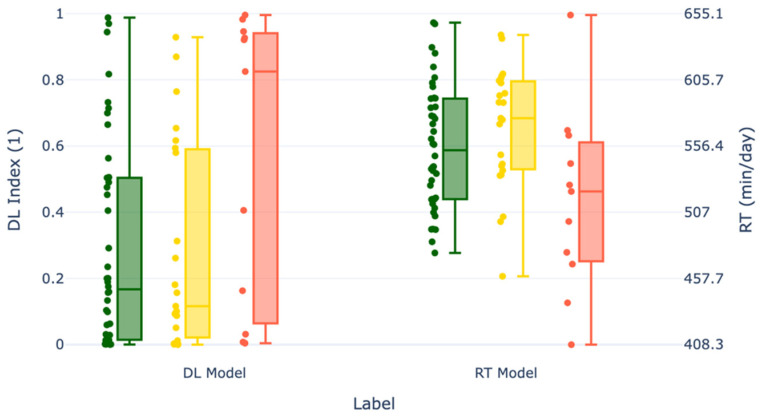
Boxplots of maximum index of the DL model until 2 h postpartum and average RT (RT Model) at day −1 (green) grouped by healthy (iCa > 1.05 mmol/L; green), subclinical hypocalcemia (iCa 0.8–1.05 mmol/L; yellow), and low-calcium (iCa < 0.8 mmol/L; red).

**Figure A6:**
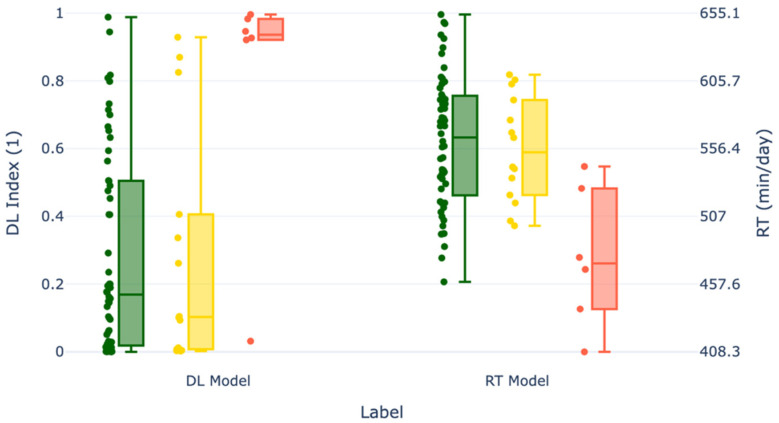
Boxplots of maximum index of the DL model until 2 h postpartum and average RT (RT Model) at day −1 grouped by healthy (tCa > 2.2 mmol/L; green), subclinical hypocalcemia (tCa 2.2–1.8 mmol/L; yellow), and low-calcium (tCa < 1.8 mmol/L; red).

## Data Availability

The datasets presented in this article are not readily available because to technical limitations. Requests to access the datasets should be directed to smaXtec animal care GmbH, 8010 Graz.

## Abbreviations

The following abbreviations are used in this manuscript:

AREC	Agricultural Research and Education Center
CNNs	convolutional neural networks
DL	deep learning
LC	Low-calcium
iCa	ionized calcium
LA	locomotion activity
LSTM	Long Short Term Memory
RCD	Reticular contraction duration
RCDV	Percentage of succesful RCD calculations
RCNR	Rumen Cycle Net Rate
RETT	reticular temperature
ROC	receiver operating chracteristic
RRCR	reticuloruminal motility
RT	rumination time
SCH	subclinical hypocalcemic
tCa	total calcium

## Appendix A

### Appendix A.1

This indicates that those metrics are well suited for hypocalcemia risk prediction with DL models.

#### Appendix A.1.2. ROC Curve Analysis Rumination Threshold Approach

The ROC curve for the mean of all relevant days antepartum (−4, −3.5, −3, −2.5, −1.5, −1, −0.5) of the RT according to tCa (healthy and LC) showed an AUC value of 0.81 (Figure A4), where the cut-off for the RT was calculated at 510.52 min/day for all relevant days before calving (Figure A4).

**Table A1:** Rumination time (RT as mean and Standard deviation), odds ratio, optimal cut-off point, specificity, sensitivity, AUC, and *p*-values for the days: −4, −3.5, −3, −2.5, −1.5, −1, −0.5, 0, 0.5, 1, 2, 2.5.

Time-Point	Mean RT Healthy (min/d)	SD RT Healthy (min/d)	Mean RT LC (min/d)	SD RT LC (min/d)	Odds Ratio	Optimal Cut Off (min/d)	Sensitivity	Specifitiy	AUC	*p*-Value
**Total serum calcium**										
**−4**	555.62	±51.09	481.14	±26.61	42.47	510.52	100	77.42	0.91	0.002
**−3.5**	555.77	±38.98	496.38	±48.23	15.69	549.28	100	54.84	0.83	0.005
**−3**	551.53	±47.62	499.73	±56.89	6.86	524.10	66.67	77.42	0.75	0.034
**−2.5**	547.72	±51.29	474.25	±59.97	8.33	510.21	66.67	80.65	0.74	0.032
**−1.5**	550.99	±48.62	501.60	±49.22	10.40	503.17	66.67	83.87	0.77	0.039
**−1**	561.51	±49.53	477.27	±51.34	60.00	480.00	66.67	96.77	0.87	<0.001
**−0.5**	570.73	±53.84	491.57	±57.88	60.00	496.75	66.67	96.77	0.86	0.005
**0**	558.24	±64.29	476.35	±79.17	10.50	540.53	83.33	67.74	0.80	0.008
**+0.5**	475.16	±66.33	349.77	±112.72	33.75	410.00	83.33	87.10	0.83	<0.001
**+1**	473.02	±55.59	377.32	±60.55	33.75	416.10	83.33	87.10	0.89	0.001
**+2**	574.63	±62.65	510.31	±53.85	13.50	523.46	66.67	87.10	0.78	0.039
**+2.5**	583.36	±59.04	523.78	±44.33	33.75	533.12	83.33	87.10	0.81	0.045
**Ionized serum calcium**										
**+0.5**	455.33	±66.87	385.60	±100.86	6.44	417.71	72.73	70.73	0.71	0.013
**+1**	450.38	±56.06	403.22	±54.72	5.75	445.09	81.82	56.10	0.73	0.044

#### Appendix A.1.3. Association of Parameter Measured by Reticuloruminal Bolus to Periparturient Ca-Concentrations

On the relevant days before parturition, weak (r = 0.1 to 0.3) to moderately strong (r = 0.3 to 0.5) positive correlation coefficients were found between the rumination time and the peripartal tCa concentration (Figure 2). The *p*-values were below the significance level of 0.05. while there were no significant correlations with RRCR (*p* > 0.08) and RETT (*p* > 0.1) before calving.

#### Appendix A.1.4. Temperature Decrease Events vs. [iCa] and [tCa]

In the smaXtec applications, temperature decrease events are triggered when an anomaly in the decrease in RETT is detected. As can be seen in Table A2 and Table A3, LC cows are most likely to experience temperature decrease events, followed by SCH, with healthy cows having the lowest percentage of temperature decrease events. This is true for both classification by [iCa] (Table A2) and [tCa] (Table A3).

**Table A2:** Number of cows with temperature decrease event after calving and percentage of cows with temperature decrease event for each class according to iCa measurements.

Percentage	Nr. of Cows with Temp. Decrease	Nr. of Cows	Label
54.5	6	11	LC
34.8	8	23	SCH
28.6	12	42	healthy

**Table A3:** Number of cows with temperature decrease event after calving and percentage of cows with temperature decrease event for each class according to tCa measurements.

Percentage	Nr. of Cows with Temp. Decrease	Nr. of Cows	Label
66.7	4	6	LC
50	7	14	SCH
26.8	15	56	healthy

#### Appendix A.1.5. Index Values Against Ca Concentration

Both the DL and RT approaches show clear differences between healthy and LC cows (cf. Figure A5 and Figure A6). The DL model’s prediction index shows clearer group separation for LC cows than the RT-based approach, particularly for classifications based on tCa (Figure A6).
